# Author’s Reply

**Published:** 2010

**Authors:** Dale Birenbaum

**Affiliations:** Chairman and Residency Program Director, Florida Hospital Emergency Medicine Residency Program, Orlando, FL, USA

Sir,

Thank you for your excellent observations[1] in our article.[2]

The goal of the paper was to clarify and stimulate further discussion with colleagues in order to improve care in the clinical arena. As stated, it is generally agreed that hypoglycemia should be treated in patients with acute ischemic stroke with the goal to achieve normoglycemia. Marked elevations of blood glucose levels should be avoided.

It has taken well over 14 years for the stroke community to embrace treating ischemic stroke patients within the 3-h window. The recent ECASS-III trail results suggest that the window for intravenous thrombolytics may be extended to 4.5 h after the onset of stroke symptoms. Let me reemphasize that in all instances, having more time does not meant that we should waste it, because patients will have better outcomes if they are treated earlier.

When it comes to imaging, the American College of Radiology (ACR) continually updates its guidelines for imaging pathways through the ACR Appropriateness Criteria.[3] Dynamic computed tomography (CT) and magnetic resonance imaging (MRI) scanning will have a significant impact on shaping the future of modern stroke care.

The ACR prefers MRI to CT for acute stroke and while some sequences may be obtained quickly, it is not currently available and practical for most centers. The recommended MRI sequences are T1, T2, fluid-attenuated inversion recovery sequence, GRE (for blood), diffusion-weighted imaging (DWI) for acute ischemia, MRA and PWI (for penumbra imaging). When available, it is not uncommon in the ED to use DWI-MRI when the diagnosis of an acute stroke is unclear as it can help confirm the diagnosis.[4]
